# Therapeutic application of CCK2R-targeting PP-F11: influence of particle range, activity and peptide amount

**DOI:** 10.1186/s13550-014-0047-1

**Published:** 2014-08-30

**Authors:** Mark W Konijnenberg, Wout A P Breeman, Erik de Blois, Ho Sze Chan, Otto C Boerman, Peter Laverman, Petra Kolenc-Peitl, Marleen Melis, Marion de Jong

**Affiliations:** Department of Nuclear Medicine, Erasmus MC, PO Box 2040, 3000 CA Rotterdam, The Netherlands; Department of Nuclear Medicine, Radboud University Medical Center, 6525 GA Nijmegen, The Netherlands; Department of Nuclear Medicine, University Medical Centre Ljubljana, SI-1525 Ljubljana, Slovenia; Department of Radiology, Erasmus MC, 3000 CA Rotterdam, The Netherlands

**Keywords:** Preclinical radionuclide dosimetry, Radiobiology, Tumour cure model, Specific activity, CCK2 peptide receptor saturation, Y-90, Lu-177, Bi-213, Minigastrin

## Abstract

**Background:**

Targeted radionuclide therapy with high-energy beta-emitters is generally considered suboptimal to cure small tumours (<300 mg). Tumour targeting of the CCK2 receptor-binding minigastrin analogue PP-F11 was determined in a tumour-bearing mouse model at increasing peptide amounts. The optimal therapy was analysed for PP-F11 labelled with ^90^Y, ^177^Lu or ^213^Bi, accounting for the radionuclide specific activities (SAs), the tumour absorbed doses and tumour (radio) biology.

**Methods:**

Tumour uptake of ^111^In-PP-F11 was determined in nude mice bearing CCK2 receptor-transfected A431 xenografts at 1 and 4 h post-injection for escalating peptide masses of 0.03 to 15 nmol/mouse. The absorbed tumour dose was estimated, assuming comparable biodistributions of the ^90^Y, ^177^Lu or ^213^Bi radiolabelled peptides. The linear-quadratic (LQ) model was used to calculate the tumour control probabilities (TCP) as a function of tumour mass and growth.

**Results:**

Practically achievable maximum SAs for PP-F11 labelled with ^90^Y and ^177^Lu were 400 MBq ^90^Y/nmol and 120 MBq^177^Lu/nmol. Both the large elution volume from the 220 MBq ^225^Ac generator used and reaction kinetics diminished the maximum achieved ^213^Bi SA in practice: 40 MBq ^213^Bi/nmol. Tumour uptakes decreased rapidly with increasing peptide amounts, following a logarithmic curve with ED_50_ = 0.5 nmol. At 0.03 nmol peptide, the (300 mg) tumour dose was 9 Gy after 12 MBq ^90^Y-PP-F11, and for ^111^In and ^177^Lu, this was 1 Gy. A curative dose of 60 Gy could be achieved with a single administration of 111 MBq ^90^Y labelled to 0.28 nmol PP-F11 or with 4 × 17 MBq ^213^Bi (0.41 nmol) when its α-radiation relative biological effectiveness (RBE) was assumed to be 3.4. Repeated dosing is preferable to avoid complete tumour receptor saturation. Tumours larger than 200 mg are curable with ^90^Y-PP-F11; the other radionuclides perform better in smaller tumours. Furthermore, ^177^Lu is not optimal for curing fast-growing tumours.

**Conclusions:**

Receptor saturation, specific radiopharmaceutical activities and absorbed doses in the tumour together favour therapy with the CCK2 receptor-binding peptide PP-F11 labelled with ^90^Y, despite its longer β-particle range in tissue, certainly for tumours larger than 300 mg. The predicted TCPs are of theoretical nature and need to be compared with the outcome of targeted radionuclide experiments.

## Background

PP-F11, a minigastrin analogue, was identified to be one of the most promising cholecystokinin 2 (CCK2) receptor-binding peptides to target medullary thyroid cancer (MTC) and small cell lung cancer lesions in a comparative search by the EU COST Action BM0607 [1]. Low retention in the kidneys by PP-F11 could be achieved by replacement of the gastrin pentaglutamic acid sequence in minigastrins with five d-glutamic acid residues. Indeed in nude mice, ^111^In-PP-F11 showed low retention in the kidneys in combination with a high uptake in A431 xenografts transfected with the CCK2 receptor in comparison with other minigastrin analogues. The tumour and physiological organ uptake was measured at 1 and 4 h after injection of 370 kBq/0.03 nmol ^111^In-labelled PP-F11. Moreover, *in vitro* cell uptake experiments showed a high affinity of PP-F11 for the CCK2 receptor and a high internalisation rate into CCK2 receptor-transfected A431 cells [2]. The next step towards clinical application of peptide receptor radionuclide therapy (PRRT) using this compound is a PRRT study in mice.

Preclinical PRRT studies are typically being performed in mice with tumours bearing an initial size of 8 to 9 mm in diameter, which corresponds to a spherical volume of 270 to 380 mm^3^, but due to different growth patterns, tumours in different animals may show large variation in size.

Low-energy beta-emitting radionuclides, such as ^177^Lu and ^131^I, are considered favourable to maximise the absorbed dose in tumours of this small size. For pragmatic reasons, the choice for a therapeutic radionuclide is often between the beta-emitters ^90^Y and ^177^Lu, as both are readily available. The latter choice (^177^Lu) seems to be optimal, as the mean beta-energy of 0.133 MeV yields a high probability of cure in the 1- to 3-mm (0.5 to 14 mm^3^) size range [3]. The high-energy beta-emitter ^90^Y (0.933 MeV) shows optimal tumour control in the 28- to 42-mm range. Neither ^213^Bi nor any other α-emitter was evaluated in the paper by O'Donoghue et al., but with the availability of ^225^Ac generators [4] the short-range emitter ^213^Bi has become a very interesting radionuclide for PRRT as well.

The theoretical prediction of [3] was confirmed in preclinical studies of somatostatin receptor-mediated PRRT comparing the therapeutic effects of ^177^Lu-DOTA,Tyr^3^-octreotate (DOTAtate) and ^90^Y-DOTA,Tyr^3^-octreotide (DOTAtoc) in large and small tumours implanted in rats [5,6]. With ^90^Y, the ideal tumour size for cure was found to be 3 to 9 cm^2^ (length × width, product of the two largest perpendicular diameters), which corresponds to spherical tumours with diameters between 20 and 34 mm and masses between 4 and 20 g. The absorbed dose to these tumours was between 48 and 60 Gy. With ^177^Lu, a more favourable response rate was found in rats bearing smaller tumours (size <1 cm or mass <0.5 g) compared with those bearing larger tumour masses. The absorbed dose to these small tumours was about 58 Gy.

We reasoned for the PRRT study that the ideal β-emitting radionuclide for labelling PP-F11 should be selected taking into account not only the absorbed radiation dose, but also the relation between the uptake kinetics in various sizes of tumours and the peptide amount to be injected. The specific activity achievable for each radionuclide dictates the peptide amount to be injected for a tumour radiation dose of 60 Gy; tumour cure is not always possible with the same peptide amount of 0.03 nmol (corresponding to 0.1 μg) as was used in the biodistribution study. Especially, neutron capture reactor-produced radionuclides show a limitation in the maximum achievable specific activity [7]. The specific activity of the peptide should be optimized to achieve the highest possible absorbed dose to the tumour taking both the peptide mass with maximum receptor-mediated uptake and the maximum achievable specific activity into account. It is well known that unlabelled peptide will compete with the labelled peptide for the limited number of receptors and thereby reduce the uptake in target tissue [8-11].

Estimation of the optimal therapy setting has been performed in this study for preclinical testing of PP-F11, assumed to be labelled with either ^90^Y, ^177^Lu or ^213^Bi. The absorbed dose in small tumours was determined, assuming the same biodistribution profile for all radiolabelled analogues and taking the influence of peptide amount on its pharmacokinetics into account as well as the radiation transport of the emitted spectra. The aim of this study was to determine which radionuclide is capable of reaching tumour-sterilizing absorbed radiation doses to the tumour of at least 60 Gy, as such a dose is expected to lead to a high probability of tumour cure over a wide range of tumour sizes according to the linear-quadratic (LQ) model (from 1 to 500 mm^3^) [3,6]. For this purpose, the most important parameters influencing the dose-effect relation for curing tumours have to be determined. In this study, only the mean absorbed dose to the tumour was taken into account, without correction for possible microdosimetry induced enhancements of the absorbed dose effects by high linear energy transfer (LET) spectra and/or bystander effects.

## Methods

### Peptide synthesis

The synthesis of the peptide PP-F11 has been described before by Kolenc-Peitl et al. [1,12]. In summary, PP-F11 was synthesized using standard Fmoc-based solid-phase peptide synthesis and conjugated with 1,4,7,10-tetraazacyclododecane-1,4,7,10-tetraacetic acid (DOTA), using one of the carboxylic groups of DOTA. The peptide structure was confirmed by mass spectrometry and NMR. The amino acid sequence of PP-F11 is DOTA-DGlu-DGlu-DGlu-DGlu-DGlu-DGlu-Ala-Tyr-Gly-Trp-Met-Asp-Phe-NH2, and its molecular weight is 2,049 g/mol.

### Radiolabelling

DOTA-conjugated peptides were radiolabelled with ^111^In by incubation with ^111^InCl_3_ (Covidien, Petten, The Netherlands) in 0.25 M ammonium acetate buffer, pH 5.0 under strict metal-free conditions. To reduce oxidation of the peptides during radiolabelling, 100 μg (10 μl) selenomethionine was added. The labelling mixture was incubated for 30 min at 95°C. After incubation, 50 mM EDTA was added to a final concentration of 5 mM. Radiochemical purity and the presence of oxidized peptide were checked on an HPLC system (1200 series LC system; Agilent Technologies, Palo Alto, CA, USA) equipped with an Alltima RP-C18 column (5 μm, 4.6 × 250 mm; Alltech, Deerfield, IL, USA) and an in-line NaI radiodetector (Raytest, Straubenhardt, Germany). A gradient from 0.1% trifluoroacetic acid in water to 0.1% trifluoroacetic acid in acetonitrile was used. The maximum specific activity of the peptide was 11 GBq/μmol.

### Biodistribution studies

Biodistribution and tumour targeting of the ^111^In-labelled PP-F11 were studied in female athymic BALB/c mice with subcutaneous tumours induced by inoculation of A431 cells. Mice were inoculated with 2 × 10^6^ A431-CCK2R cells (0.2 ml) in the left flank and with A431 mock-transfected cells in the right flank. After approximately 10 days, when the tumours had reached a weight of approximately 100 to 200 mg, the mice were randomly divided into groups of five mice. The mice were injected intravenously with 370 kBq (0.03 nmol) of ^111^In-labelled peptide via the lateral tail vein. In an additional experiment, six groups of mice were injected with 370 kBq/0.05 nmol ^111^In-PP-F11 supplemented with an increasing amount (range: 3, 10, 30, 100, and 300 times) of molar excess of the unlabelled PP-F11 to determine the receptor saturation effect as well as the non-receptor specific uptake in CCK2 receptor-expressing tissues. Mice were killed by CO_2_ asphyxiation at 1 and 4 h after administration in the biodistribution study and after 2 h in the receptor saturation study. A blood sample was drawn, and organs of interest and the tumour were dissected, weighed and counted in a gamma counter. The animal experiments were approved by the local animal welfare committee and performed according to national regulations.

### Dosimetry

#### Tumour absorbed dose rate *S*-factors

Absorbed fractions of energy emitted from ^90^Y, ^111^In, ^177^Lu, ^213^Bi and its daughters were determined in a spherical geometry using the Monte Carlo code MCNP5 (version 1.4 [13]). The mean absorbed energies were calculated for spherical tumours of 1, 10, 100, 200, 300 and 500 mg containing ICRU-44 tissue [14] assuming an average tissue mass density of 1 g/ml. The emission spectra were taken from the Medical Internal Radiation Dose (MIRD) radionuclide data and decay schemes book [15]. The main characteristics of the emissions by ^90^Y, ^111^In, ^177^Lu, ^213^Bi and its daughters ^213^Po, ^209^Pb and ^209^Tl are listed in Table 1. Electrons and photons with an energy below 1 keV were assumed to be absorbed at the site of emission. The total energy emitted by electrons below 1 keV in the spectra from ^111^In and ^177^Lu is added to the absorbed energy by the higher energy electrons. The default settings for electron and photon physics within MCNP were used, with the exception of the electron energy straggling logic, where the option ‘DBCN 17J 2’ was used to be independent of pre-set energy boundaries [16,17]. For each component in the emission spectra, 10 million histories were calculated to keep the statistical variance below 5%.

**Table 1:** **Emission spectra and decay half-lives of** ^**90**^ **Y,** ^**111**^ **In,** ^**177**^ **Lu and** ^**213**^ **Bi and its daughters**

**Radionuclide**	**Decay** ***T***_**1/2**_	**Radiation**	**Yield (/decay)**	***E***_**av.**_ **(MeV)**	***E***_**max**_ **(MeV)**
^90^Y	64.1 h	β^−^	1	0.9329	2.2801
^111^In	67.3 h	γ	0.906	0.1713	
		γ	0.941	0.2454	
		Auger e^−^ <1 keV	6.26	0.000159	
		Electrons >1 keV	1.33	0.0255	
		X-rays <1 keV	8.60	0.000017	
		X-rays >1 keV	0.901	0.0220	
^177^Lu	159.5 h	β^−^	1	0.1333	0.4978
		γ	0.064	0.1129	
		γ	0.11	0.2084	
		Auger e^−^ <1 keV	0.819	0.000138	
		Electrons >1 keV	0.452	0.0322	
^213^Bi	45.59 min	α	0.00155	5.55187	
		α	0.01935	5.86959	
		β^−^	0.9791	0.4339	1.4220
		γ	0.261	0.44046	
		Auger e^−^ <1 keV	0.474	0.000113	
		Electrons >1 keV	0.152	0.1261	
^213^Po	4.2 μs	α	1	8.38	
^209^Pb	3.253 h	β^-^	1	0.1974	0.6444
^209^Tl	2.161 min	γ	0.998	1.57	
		γ	0.969	0.465	
		γ	0.843	0.117	
		Auger e^−^ <1 keV	2.64	0.000108	
		Electrons >1 keV	0.830	0.0384	

Energies are indicated for the beta ray (mean and maximum), Auger electrons, low-energy electrons and X-ray spectra. Below the cut-off value of 1 keV, all energy is assumed to be absorbed at the site of emission. Only gamma rays with an emission yield of more than 0.01/decay are indicated.

The absorbed energy distribution was determined in each spherical tumour by using 25 equal-volume concentric spherical shells within the source volume and 25 equal-volume spherical shells around the tumour extending to a radial distance of 1 cm as a consistency check of the electron transport model used. Absorbed dose rate per unit activity *S*-factors was calculated according to the equation: $S=k\frac{{\displaystyle \sum_i{y}_i{E}_i{\phi}_i}}{m},$ where *k* is a conversion factor to dose, *ϕ*_*i*_ is the absorbed fraction of emitted energy *E*_*i*_ that is emitted with yield *y*_*i*_, and *m* is the mass of the spherical shell part. The dose to the tumour is calculated according to the MIRD scheme by the product of the cumulated activity *Ã* and the absorbed dose *S*-factor *D* = *Ã* × *S* = *A*_0_ × *ã* × *S*, where *A*_0_ is the administered activity and *ã* is the time-integrated activity coefficient (TIAC; formerly indicated by residence time *τ*) of the radioactivity in the tumour [18].

#### Influence of peptide kinetics and receptor saturation on dosimetry

**Figure 1:**
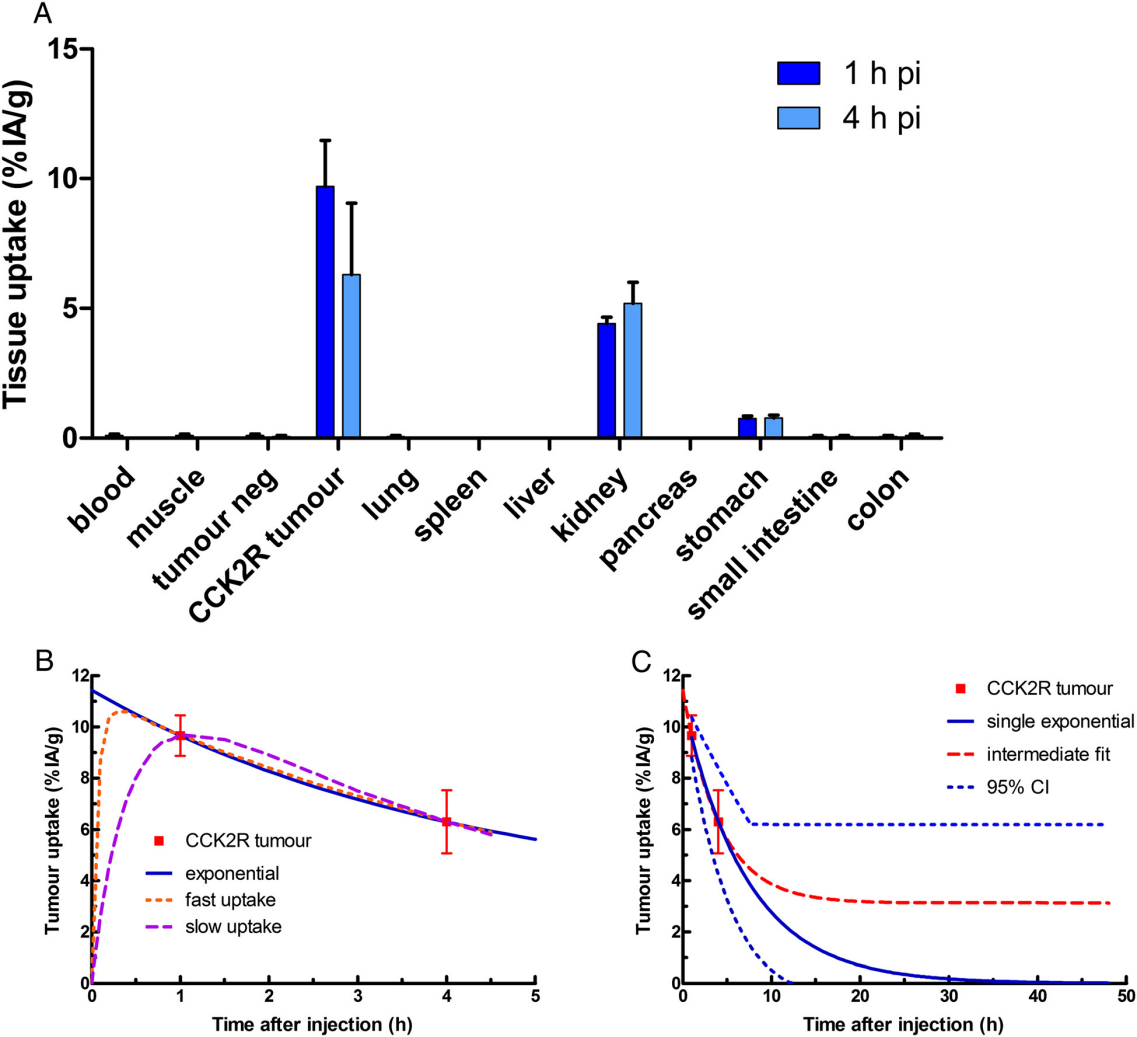
**Biodistribution and kinetic profile. (A)** Biodistribution of 0.03 nmol ^111^In-labelled PP-F11 in nude mice with CCK2R-transfected A431 tumour xenografts at 1 and at 4 h (reproduced from [1]). **(B)** Kinetic profile of radioactivity uptake in the CCK2 receptor-positive tumour xenograft with three options that are considered for the uptake phase: single-exponential, fast uptake with *T*_1/2_ of 2.7 min and slow uptake with *T*_1/2_ of 17 min. **(C)** Variance in kinetic profile of the radioactivity clearance from the tumour: the solid blue curve shows the single exponential with *T*_1/2_ of 4.9 h, the middle dashed red curve shows the double exponential with final clearance *T*_1/2_ of 150 h and the blue dotted curves show the 95% confidence interval for the tumour clearance.

The cumulated activity in the tumour *Ã* is based on the ^111^In-PP-F11 biodistribution data at 1 and 4 h for a peptide mass of 0.03 nmol (0.1 μg) (Figure 1A) [5]. In mice with s.c. CCK2 receptor-expressing A431 tumours, the radiolabelled PP-F11 showed a high tumour uptake and retention (9.7 ± 1.8 and 6.3 ± 2.8%ID/g, 1 and 4 h p.i.), which was among the highest of the 12 CCK2 analogues considered [1]. Based on the two time points, two possible biodistribution patterns were applied to evaluate the consequence of different kinetic profiles. The first approach was a mono-exponential curve with a biological clearance half-life of 4.9 ± 2.3 h (Figure 1B). For the second approach, an additional slower clearance compartment was assumed with a clearance half-life of 150 h, being the median curve between the single exponential and the situation with just physical decay after 4 h, indicated in Figure 1C. As not only the clearance kinetics, but also the uptake kinetics will influence the cumulated activity, especially for the shortest half-life radionuclide ^213^Bi (Figure 1B), additionally the effect of both slow and fast uptake kinetics was considered.

The *in vivo* behaviour of PP-F11 labelled with ^90^Y, ^177^Lu or ^213^Bi was assumed to be equivalent to that of ^111^In-labelled PP-F11.

The tumour uptake concentration data ([*B*]) as a function of peptide mass (*M*_p_) was fitted to the sigmoid-shaped one-site competition curve:

1 $$ \left[B\left({M}_{\mathrm{p}}\right)\right]=\left[{B}_{\mathrm{unsp}}\right]+\frac{\left[{B}_{\mathrm{spec}}\right]}{1+\frac{M_{\mathrm{p}}}{{\mathrm{ED}}_{50}}}, $$

where [*B*_unsp_] is the unspecific-bound concentration asymptote at high peptide mass, [*B*_spec_] is the receptor-bound concentration, and ED_50_ is the peptide mass that lowers the tumour concentration by 50%. This curve gives an indication of the limited number of receptors available.

#### Activity for 60-Gy tumour dose

The amount of activity needed with each radionuclide to obtain a radiation dose of 60 Gy in a 300-mg tumour is derived for the dosimetry according to the 0.03 nmol peptide mass biodistribution. The dosimetry is adjusted according to the peptide mass needed to label the peptide at a practically achievable specific activity using the logarithmic receptor saturation function. Traditionally, the activity *A*_0_(*M*_p_) needed to obtain an absorbed dose *D* in a tumour with mass *m* and using the biodistribution for a peptide mass of *M*_p_ = 0.03 nmol is obtained by:

2 $$ {A}_0(0.03)=\frac{D}{{\overset{\sim }{a}}_mS\left(m\leftarrow m\right)}. $$

The relation for *A*_0_(*M*_p_) as a function of the peptide mass *M*_p_ changes for the maximum (achievable) specific activity *A*_sp_ at both sides of the equation:

3 $$ {A}_0\left({M}_{\mathrm{p}}\right)={M}_{\mathrm{p}}\times {A}_{\mathrm{sp}}=\frac{\left[B(0.03)\right]D}{\left[B\left({M}_{\mathrm{p}}\right)\right]{\overset{\sim }{a}}_mS\left(m\leftarrow m\right)}. $$

This equation can be solved to obtain the amount of peptide mass *M*_p_ needed with specific activity *A*_sp_ that will deliver a dose *D* in a tumour with mass *m*. Not only the absorbed dose but also the amount of peptide, the specific activity used and the total number of receptor binding sites available are of importance. It is assumed the shape of the kinetics does not change with peptide mass, just the uptake value itself. The maximum and practically achievable specific activities for labelling peptides with ^111^In, ^90^Y and ^177^Lu are indicated in Table 2, as obtained from Breeman et al. [7]. Maximum specific activity is reached when 1 nmol of DOTA incorporates 1 nmol of radionuclide. In practice, the specific activity is severely hampered by the reaction kinetics and challenged uptake by contaminants in the radionuclide solution, either from target material or from radioactive decay products. The highest practically achievable specific activity is obtained with ^111^In, and ^90^Y shows an almost four times higher specific activity (SA) than ^177^Lu. The administered amount of peptide per injection was maximised at 0.5 nmol (1.02 μg) to stay within practically feasible limits in both volume and radioactivity. The practical limit in specific activity of ^213^Bi-labelled peptides was obtained at our institute with a 220 MBq ^225^Ac generator.

**Table 2:** **Production methods and specific activities (SA) of** ^**90**^ **Y,** ^**111**^ **In,** ^**177**^ **Lu and** ^**213**^ **Bi, both per nmol PP-F11 with 2,049 g/mol**

	^**90**^ **Y**	^**111**^ **In**	^**177**^ **Lu**	^**213**^ **Bi**
Production route	Elution from ^90^Sr generator	Cyclotron ^112^Cd(p,2n)^111^In	Reactor ^176^Lu(n,γ)^177^Lu	Elution from ^225^Ac generator
Maximum SA (GBq/nmol)	1.8	1.7	0.73	153
Practical SA (MBq/nmol)	400	800	120	40

The practical achievable SA of ^213^Bi with peptide is much lower than after labelling with ^90^Y, ^111^In or ^177^Lu, due to the at least twice higher reaction volume with ^213^Bi. The elution volume of the generator is fixed at approximately 600 μL, dependent on the generator bed volume in the preclinical setting using a 220 MBq ^225^Ac generator. Generators containing higher amount of ^225^Ac activity have larger bed volumes and higher elution volumes [4,19]. High labelling volume or low concentration of the labelled compounds results in poor reaction kinetics of the labelling, which can be solved by increasing the temperature or taking a longer reaction time. However, at a very high temperature, denaturation of biological materials might occur, and in the case of ^213^Bi, a longer reaction time will result in decay of activity. By reducing the elution volume and reaction time of ^213^Bi labelling, a higher SA can be achieved. Presently, the time between elution and injection in the animal is in the order of 30 min, by which already 36% of the initial ^213^Bi radioactivity has decayed.

#### Tumour control probability model

The suitability of the radionuclide for treating a tumour xenograft is investigated by constraining the tumour control probability (TCP) to at least 90% according to [3,20,21]:

4 $$ \mathrm{TCP} = \exp \left(-{N}_{\mathrm{clonogens}}S(D)\right), $$

$$ \mathrm{with}\ S(D)= \exp \left(-\alpha D\left(1+\frac{G}{\left(\frac{\alpha }{\beta}\right)}\times \frac{D}{N}\right)\right), $$

$$ \begin{array}{l}\mathrm{with}\ G\\ {}=\left\{\begin{array}{ll}\frac{T_{\mu }}{T_{\mu }+{T}_{\mathrm{eff}}}\hfill & \left(4\mathrm{a}\right)\hfill \\ {}\frac{\frac{T_{\mu }}{T_{\mu }+{T}_1}{\left({T}_1{A}_1\right)}^2+2\frac{T_{\mu }{T}_1{T}_2\left({T}_{\mu }{T}_1+{T}_{\mu }{T}_2+{T}_1{T}_2\right)}{\left({T}_{\mu }+{T}_1\right)\left({T}_{\mu }+{T}_2\right)\left({T}_1+{T}_2\right)}{A}_1{A}_2+{\frac{T_{\mu }}{T_{\mu }+T}}_2{\left({T}_2{A}_2\right)}^2}{{\left({T}_1{A}_1+{T}_2{A}_2\right)}^2}\hfill & \left(4\mathrm{b}\right)\hfill \end{array}\right.\end{array} $$

The survival *S* of clonogenic cells as a function of dose *D* in the tumour is described by the linear-quadratic model. The dose per fraction *D*/*N* is used in the quadratic term to allow correction for fractionation of the therapy. The dose prolongation or Lea-Catcheside factor *G* is used to adjust the quadratic part of the LQ model for radionuclide-induced dose rates. During the exposure with effective half-life *T*_eff_, sub-lethal damage is being repaired according to an exponential function with repair half-life *T*_μ_. The single-exponential clearance with effective half-life *T*_eff_ is indicated in Equation 4a for *G*. Equation 4b is for a bi-exponential clearance curve with effective half-lives *T*_1_ and *T*_2_ and amplitudes *A*_1_ and *A*_2_. The second expression for *G* (4b) can be modified to take the uptake kinetics into account, with *A*_2_ = −*A*_1_ and $A(t)={A}_1\left({e}^{-{\lambda}_1t}-{e}^{-{\lambda}_2t}\right),$ which leads to

5 $$ G=\frac{T_{\mu}\left({T}_1{T}_{\mu }+{T}_2{T}_{\mu }+{T}_1{T}_2\right)}{\left({T}_1+{T}_2\right)\left({T}_1+{T}_{\mu}\right)\left({T}_2+{T}_{\mu}\right)}. $$

In this work, the clonogenic cell density is assumed to be 10% of the total cell density of 10^9^/g. The following LQ model parameters were considered: *α* = 0.27 Gy^−1^, *α*/*β* = 6 Gy and *T*_μ_ = 0.5 h (squamous cells, see [22]). The repair half-life of 0.5 h is in the lower range for tumour sub-lethal repair half-lives [23].

#### Effect of tumour growth on TCP

Correction of TCP for tumour growth with doubling time *T*_D_ during the dose accumulation was made by including the tumour growth in the LQ model equation for cell survival:

6 $$ S\left(D,T\right)= \exp \left(-\alpha D(T)\left(1+\frac{G}{\frac{\alpha }{\beta }}\times \frac{D(T)}{N}\right)+\gamma T\right), $$

where *γ* = ln(2)/*T*_D_ and *T* is the irradiation time. Overall, the TCP is adjusted by taking the power with *e*^*γT*irr^ the growth of the tumour during the effective irradiation time *T*_irr_ when the slope, or derivative, of the cell survival curve remains positive, as the radiation effect outweighs the tumour growth [24]. The tumour doubling time for the A431-CCK2R cells was determined to be 4 days [25]. Only the absorbed dose delivered to the tumour before the irradiation time *T*_irr_ has passed can be considered effective in cell kill. Therefore, both the absorbed dose and the TCP were corrected for the efficacious dose *D*_eff_ and the tumour growth during the irradiation time, with:

7 $$ {D}_{\mathrm{eff}}=S\left(m\leftarrow m\right){\displaystyle {\int}_{T_0}^{T_{\mathrm{irr}}}}A(t) dt. $$

In the case that it was impossible to give curative doses with the radionuclide in one administration, a treatment interval of 24 h between subsequent injections was assumed with unaltered growth in the treatment gap in between.

## Results

### Biodistribution studies

In mice with s.c. CCK2 receptor-expressing A431 tumours, the radiolabelled PP-F11 showed a high tumour uptake and retention (9.7 ± 1.8 and 6.3 ± 2.8%ID/g, 1 and 4 h p.i.) [1]. The mono-exponential curve with a biological clearance half-life of 4.9 ± 2.3 h (Figure 1B) leads to higher cumulative activities for the shorter half-life radionuclides such as ^90^Y and ^213^Bi in comparison to ^177^Lu. An additional clearance compartment was assumed with a clearance half-life of 150 h, being a median curve between the single exponential and the situation with just physical decay after 4 h, indicated in Figure 1C. Not only the clearance kinetics but also the uptake kinetics will influence the cumulated activity, especially for the shortest half-life radionuclide ^213^Bi (Figure 1B). The effect of both slow and fast uptake on the cumulated activity was considered.

**Figure 2:**
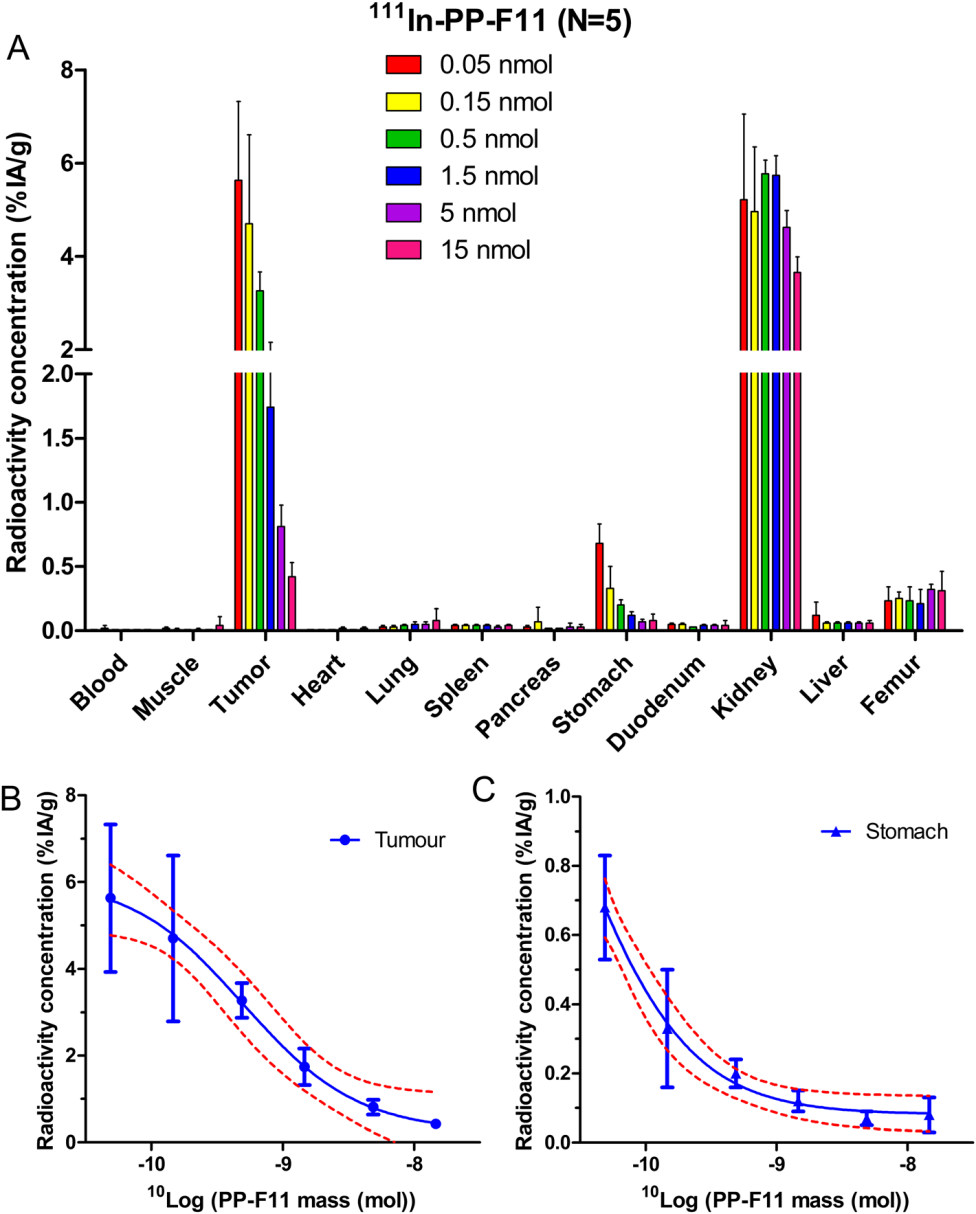
**Biodistribution and fit of displacement curve. (A)** Biodistribution of ^111^In-labelled PP-F11 with increasing excess unlabelled peptide in nude mice (*N* = 5) with CCK2R-transfected A431 tumour xenografts at 2 h after injection, showing saturation of uptake in the CCK2R tumours and stomach. **(B)** Fit of displacement radioactivity curve to the CCK2R tumour xenograft data with log(ED_50_) = −9.30 ± 0.19 (ED_50_ = 0.51 nmol (95% CI: 0.2 to 1.2), correlation *R* ^2^ = 0.80). **(C)** Fit of displacement curve to stomach uptake data with log(ED_50_) = −10.54 ± 0.38 (ED_50_ = 0.03 nmol (0.005 to 0.17), *R* ^2^ = 0.85). Error bars indicate standard deviations of the mean values.

The effect of increasing PP-F11 peptide mass on the uptake of the radiolabelled peptide in the tumour and the normal tissues is shown in Figure 2A. In the CCK2R-expressing tumour and stomach, the radioactivity uptake decreased with increasing peptide dose injected. This decrease was fitted with one-site dissociation curves (Equation 1) shown in Figures 2B,C.

### Dosimetry

#### Absorbed dose in tumours

Absorbed dose rates per MBq activity (*S*-values) were calculated for ^90^Y, ^111^In, ^177^Lu, ^213^Bi, ^213^Po, ^209^Pb and ^209^Tl in small spherical tumours and are indicated in Table 3. For ^111^In and ^177^Lu, almost the complete emitted internal conversion electron and beta-energy are absorbed in the largest sphere, whereas for ^90^Y, only 61% of the emitted energy is absorbed. In the 1-mg sphere the energy absorption *ϕ* = 10% for ^90^Y and *ϕ* = 80% and 69% for ^111^In and ^177^Lu, respectively. The absorbed energy fractions by the α-particles from ^213^Bi and ^213^Po are more than 95% in the smallest sphere and reach 100% in the 100-mg spheres.

**Table 3:** ***S*** **-values for** ^**90**^ **Y,** ^**111**^ **In,** ^**177**^ **Lu and** ^**213**^ **Bi with its daughters, in spheres with masses between 1 and 500 mg**

**Mass (mg)**	**Absorbed dose rate per radioactivity** ***S*** **-value (mGy/MBq s)**						
	^**90**^ **Y**	^**111**^ **In**	^**177**^ **Lu**	^**213**^ **Bi**	^**213**^ **Po**	^**209**^ **Pb**	^**209**^ **Tl**
1	15.4	4.66	17.0	37.6	1273	17.4	19.4
5	5.21	1.04	3.92	9.55	260	4.48	6.14
10	3.26	0.539	2.05	5.28	131	2.41	3.72
50	1.06	0.112	0.436	1.30	26.5	0.545	1.08
100	0.643	0.0576	0.222	0.695	13.3	0.281	0.643
200	0.381	0.0295	0.113	0.367	6.65	0.144	0.364
300	0.278	0.0200	0.0756	0.252	4.44	0.0971	0.258
500	0.184	0.0123	0.0457	0.156	2.67	0.0591	0.166

The radioactivity was homogeneously distributed in the tissue-like material spheres within the calculation model set up with MCNP5 v1.4 [13].

The TIACs per gram of tumour, listed in Table 4, were obtained by integration of the time-activity curves of Figure 1B,C. Non-instantaneous uptake kinetics has the most influence on the TIAC for ^213^Bi and ^213^Po, e.g. 19% difference between the single exponential and slow uptake values. Slower uptake kinetics will influence the other radionuclides by maximally 6% in single-exponential clearance and by less than 3% for the double-exponential clearance pattern, assuming linear scaling of the uptake with tumour mass delivers the mean absorbed tumour doses per administered activity as listed in Table 5. These absorbed doses, however, are only valid for 0.03 nmol PP-F11. When this amount of peptide is used, the maximum absorbed doses with the β-emitting radionuclides for both types of clearance rates are obtained with ^90^Y, e.g. for an injected activity of 12 MBq ^90^Y which can be labelled to 0.03 nmol PP-F11, the dose to a 300-mg tumour is 3 to 9 Gy for the single and the double exponential clearance rate, respectively. The absorbed doses using the other radionuclides are much lower, around 1 Gy. Therefore, multiple injections with more peptide amounts are needed to achieve 60 Gy.

**Table 4:** **Tumour time-integrated activity coefficients (TIAC) per mass for** ^**90**^ **Y,** ^**111**^ **In,** ^**177**^ **Lu and** ^**213**^ **Bi with its daughters**

**Kinetics**	**TIAC per tumour mass [in s/g]**						
	^**90**^ **Y**	^**111**^ **In**	^**177**^ **Lu**	^**213**^ **Bi**	^**213**^ **Po**	^**209**^ **Pb**	^**209**^ **Tl**
1 − Exponential	261	262	272	380	372	228	8
+ Fast uptake	258	259	269	361	353	230	8
+ Slow uptake	245	246	256	309	303	238	7
2 − Exponential	928	956	1,523	388	380	256	8

The clearance kinetics was modeled by a single-exponential (1-Exp) or by double (2-Exp) clearance (see Figure 1B,C). The uptake kinetics in the case of single-exponential clearance was either instantaneous (1-Exp) or according to the fast or slow uptake curves indicated in Figure 1B. All values are for a peptide amount of 0.03 nmol PP-F11.

**Table 5:** **Mean absorbed doses per administered activity for** ^**90**^ **Y,** ^**111**^ **In,** ^**177**^ **Lu and** ^**213**^ **Bi (including daughters) labelled to 0.03 nmol PP-F11**

**Mass (mg)**	**Absorbed dose per administered activity (mGy/MBq)**							
	^**90**^ **Y**		^**111**^ **In**		^**177**^ **Lu**		^**213**^ **Bi + daughters**	
	**1-Exp**	**2-Exp**	**1-Exp**	**2-Exp**	**1-Exp**	**2-Exp**	**1-Exp**	**2-Exp**
1	40	143	12	45	46	259	492	503
5	68	242	14	50	53	299	508	519
10	85	302	14	52	56	311	514	525
50	139	493	15	54	59	332	524	536
100	168	597	15	55	60	338	527	539
200	199	708	15	57	61	343	530	542
300	217	773	16	57	62	345	532	544
500	240	853	16	59	62	348	533	545

Tumour spheres vary in mass between 1 and 500 mg. Single-exponential (1-Exp) clearance proceeds with *T*_1/2_ = 4.8 h and double-exponential (2-Exp) clearance with *T*_1/2_ = 2.7 h (60%) and 150 h (40%).

#### Effect of peptide mass

Higher amounts of peptide are needed to guide higher amounts of radioactivity and higher absorbed doses to the tumour. However, increasing the amount of peptide will at the same time reduce the tumour TIAC, according to the saturation curve shown in Figure 2. We postulated that the kinetics of the peptide clearance were not influenced by the saturation effect, but only the final uptake at each time-point, expressed as %IA/g.

Taking the single-exponential clearance rate into account, an absorbed dose of 60 Gy in a 300-mg tumour can only be achieved by administering 5 nmol of PP-F11 peptide labelled with 2 GBq of ^90^Y or with 123 MBq ^213^Bi labelled to 0.081 nmol of PP-F11. In Table 6, an overview is listed of activities and peptide mass needed to reach an absorbed dose of 60 Gy in a 300-mg tumour for the double-exponential clearance pattern, either by single or multiple (*N*) injections with ^90^Y, ^111^In, ^177^Lu and ^213^Bi labelled to PP-F11, together with the absorbed doses in other tumour sizes, varying from 1 to 500 mg.

**Table 6:** **Activities and peptide mass needed for an absorbed dose of 60 Gy in a 300**-**mg tumour**

	^**90**^ **Y - PP-F11**		^**111**^ **In - PP-F11**	^**177**^ **Lu - PP-F11**	^**213**^ **Bi - PP-F11**
*N* × activity (MBq)	1 × 111	4 × 20	8 × 174	6 × 47	10 × 19
*N* × peptide (nmol)	0.28	4 × 0.05	8 × 0.21	6 × 0.39	10 × 0.47
Relative uptake (%)	70	97	75	62	57
Tumour mass (mg)	Mean absorbed dose to tumour (Gy)				
1	11		47	45	55
5	19		52	52	57
10	23		54	54	58
50	38		56	58	59
100	46		57	59	59
200	55		59	60	60
300	60		60	60	60
500	68		61	38	60

Multiple (*N*) injections were considered; only ^90^Y could attain 60 Gy by a single injection. The relative uptake reduction factor by the higher peptide mass is indicated. The mean absorbed doses are given for tumour spheres varying in mass between 1 and 500 mg according to the mentioned activity and assuming the double-exponential clearance with *T*_1/2_ = 2.7 h (60%) and 150 h (40%) without tumour growth. The absorbed dose for ^213^Bi was not corrected for its relative effectiveness (RBE = 1).

The TCPs for absorbed doses of 60 Gy in a 300-mg tumour are 77% for ^90^Y, 74% for ^111^In, and 48% for ^177^Lu when given in one fraction, according to Equation 4. With fractionated therapy, the TCP drops to 21% (^90^Y), 16% (^111^In) and 12% (^177^Lu), despite the same dose of 60 Gy. Both reductions in TCP are caused by the lower dose rates, either by the radionuclides' half-lives or by the fractionation scheme. The TCP for ^213^Bi PP-F11 depends on the relative biological effectiveness (RBE) of its α-radiation (93% of the absorbed dose), which is not clear yet. A RBE higher than 1.2 will already result in a TCP of more than 90% in all tumours below 300 mg. Previously, the RBE for cell killing by ^213^Bi-DOTA-octreotate has been shown to be 3.4 [26]. If this would also be the case for ^213^Bi-PP-F11, 90% TCP will already be reached with 4 × 17 MBq (0.41 nmol).

**Figure 3:**
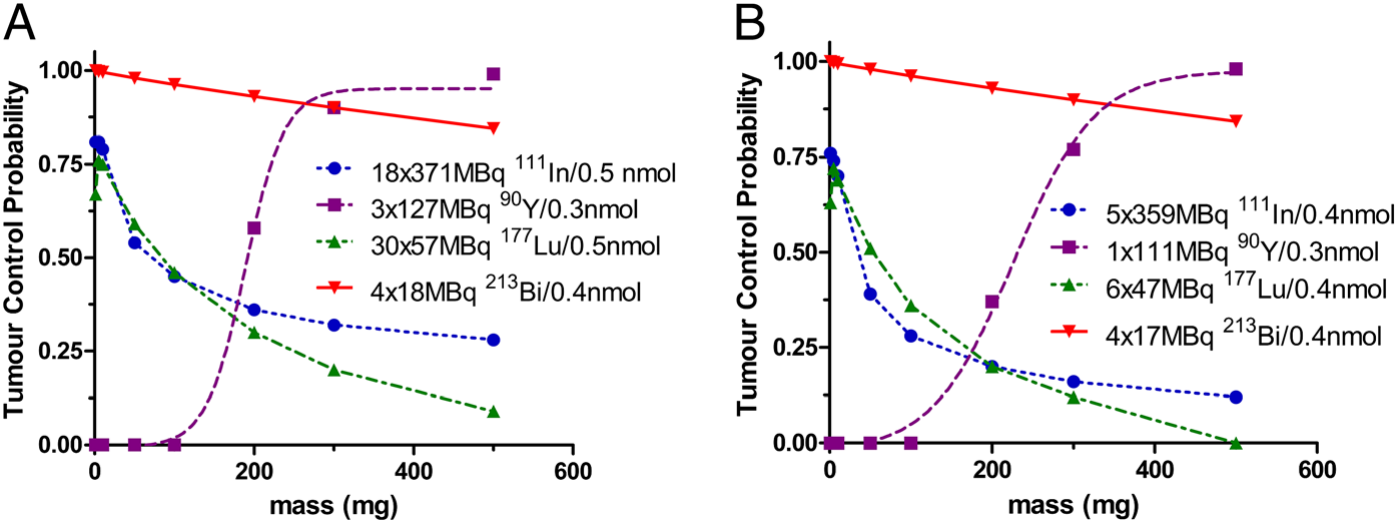
**Tumour control probability.** TCP as a function of tumour mass at activities producing a TCP of 90% in a static 300-mg tumour, or an absorbed dose of 60 Gy when lower. The clonogenic tumour cell density was 10^8^/g and the LQ model parameters were *α* = 0.27 Gy^−1^, *α*/*β* = 6 Gy and *T*_μ_ = 0.5 h. For the alpha-particle part of the dose by ^213^Bi, RBE = 3.4. **(A)** Single-exponential clearance kinetics and **(B)** double-exponential clearance kinetics.

When single-exponential clearance is assumed with consequently higher initial dose rate, the *G*-factor shows a rise: 60 Gy yields higher TCPs of 99% (^90^Y), 32% (^111^In) and 20% (^177^Lu) vs. double-exponential clearance kinetics (Figure 3). The absorbed dose needed for a TCP of 90% is 42 Gy with a single-exponential curve and single-fraction administration. For both clearance patterns, only ^90^Y and ^213^Bi are capable of delivering these curable doses, as shown in Figure 3 for the situation which does not take tumour growth into account, ^213^Bi to the whole range and ^90^Y only to the larger size tumours. Both ^111^In and ^177^Lu show the maximum TCP at small tumour size: 76% to 81% in 1 mg with ^111^In and 72% to 76% in 5 mg with ^177^Lu. The absorbed dose needed for a TCP of 90% in a 300-mg tumour with ^177^Lu is 70 Gy. The quadratic term is determinant for the TCP for the low-LET radiation, leading to higher TCPs for the single-exponential clearance pattern and to TCPs of 0 when the quadratic LQ model term β is neglected. The high amounts of total radioactivity needed to obtain cure with the β-emitters for the single-exponential clearance are not practically achievable.

The dose effect by ^213^Bi is not influenced by the quadratic term of the LQ model due to the fact that 95% of its absorbed dose is caused by high LET α-radiation without repairable damage. Because of the short half-life, the uptake kinetics, however, do show a large influence on the cure possibilities with ^213^Bi (Figure 2A). When the slow uptake kinetics (*T*_1/2_ = 16 min) are applied, the absorbed dose will be reduced by 18%, whereas for the fast uptake (*T*_1/2_ = 7 min), only a 4.2% reduction in dose is observed in comparison to the absorbed dose for instantaneous uptake. Compensation for this possible dose reduction can be obtained by administering either one additional therapy cycle or 9% more radioactivity per cycle.

#### Influence of tumour growth rate

**Figure 4:**
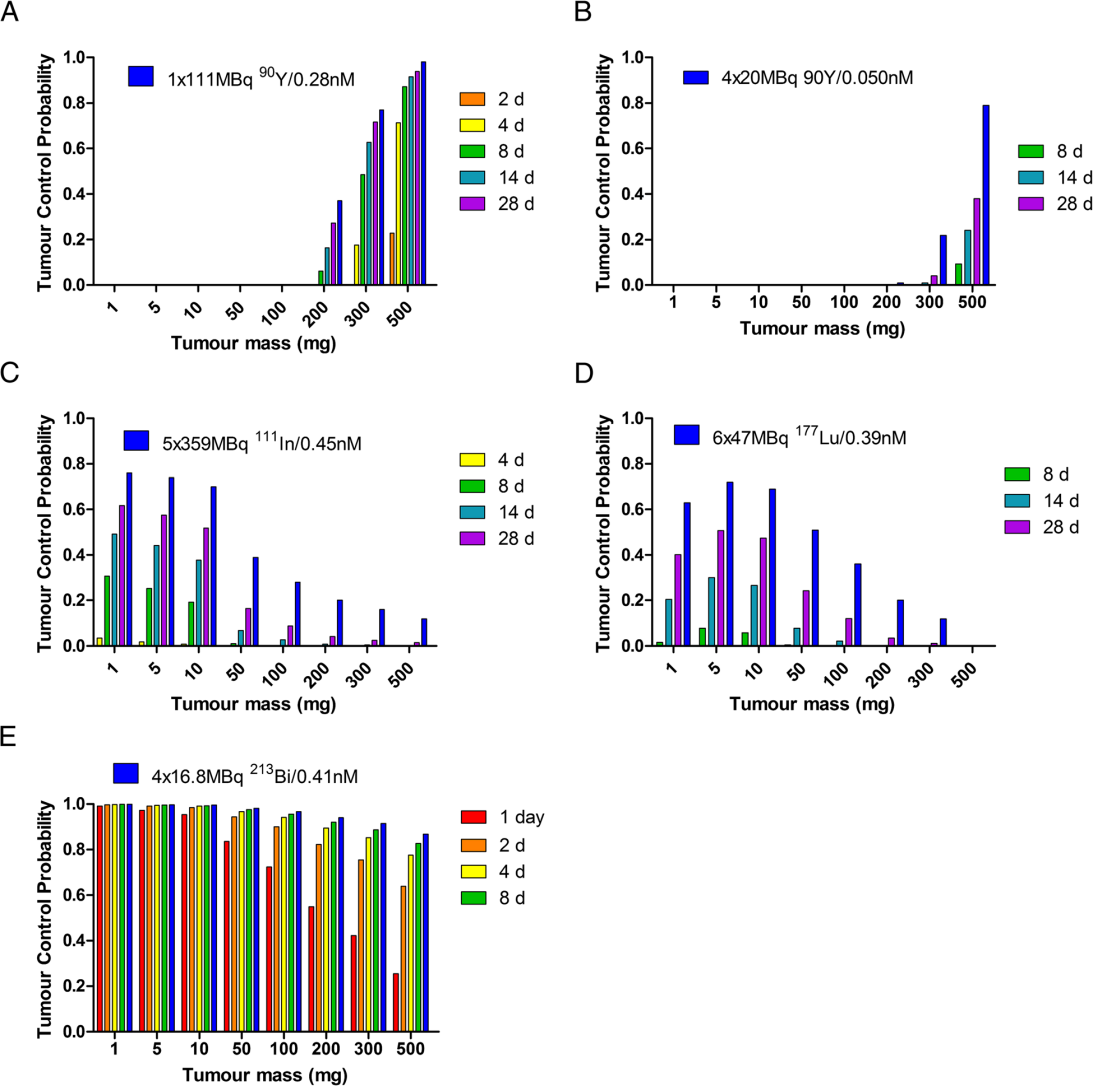
**Tumour control probability vs tumour mass.** TCP versus tumour mass for a single 111 MBq ^90^Y **(A)**, multiple administrations 4 × 20 MBq ^90^Y **(B)**, 5 × 359 MBq ^111^In **(C)**, 6 × 47 MBq ^177^Lu **(D)** and 4 × 16.8 MBq ^213^Bi **(E)** for tumour doubling times varying between 1 and 28 days, and for the static tumour, as shown in Figure 3. Only bars for TCP > 0.05 are shown, except for ^213^Bi where the 14- and 18-day doubling time results were almost equal to the static (without tumour growth) TCP shown with the blue bars.

The tumour doubling time influences the tumour cure possibilities in therapies which need the double-exponential clearance kinetics to reach therapeutic doses. The irradiation time *T*_irr_ indicates the time until the radiation cell kill outnumbers the tumour growth. With 111 MBq ^90^Y (sufficient to cure a static 300-mg tumour), *T*_irr_ can vary between 184 h (*T*_doubling_ of 2 days) and 354 h (*T*_doubling_ of 28 days). When ^90^Y is given in 4 × 20 MBq with 24-h interval, *T*_irr_ ranges between 218 and 389 h. The irradiation times for 5 × 359 MBq ^111^In are comparable to those of ^90^Y: 237 and 414 h. With 6 × 47 MBq ^177^Lu, the tumour growth takes over at later times: its irradiation times vary between 317 h (*T*_doubling_ of 2 days) and 611 h (*T*_doubling_ of 28 days). For therapy with ^213^Bi, the influence of the tumour doubling time is much less (16.8 MBq ^213^Bi: 6.7 h (for *T*_doubling_ of 2 days) and 11 h (for *T*_doubling_ of 28 days)). The build-up of the dose over this irradiation time is reduced in comparison to the build-up to infinity; for ^213^Bi, the reduction is below 0.5% and negligible like the maximal 5% reduction with ^90^Y, but with ^177^Lu, it is 18% at *T*_doubling_ of 2 days and 5% at *T*_doubling_ of 28 days. Both the effects by reduction in dose and tumour growth during the irradiation have influence on the tumour cure probabilities, as shown in Figure 4 for the double-exponential clearance.

**Figure 5:**
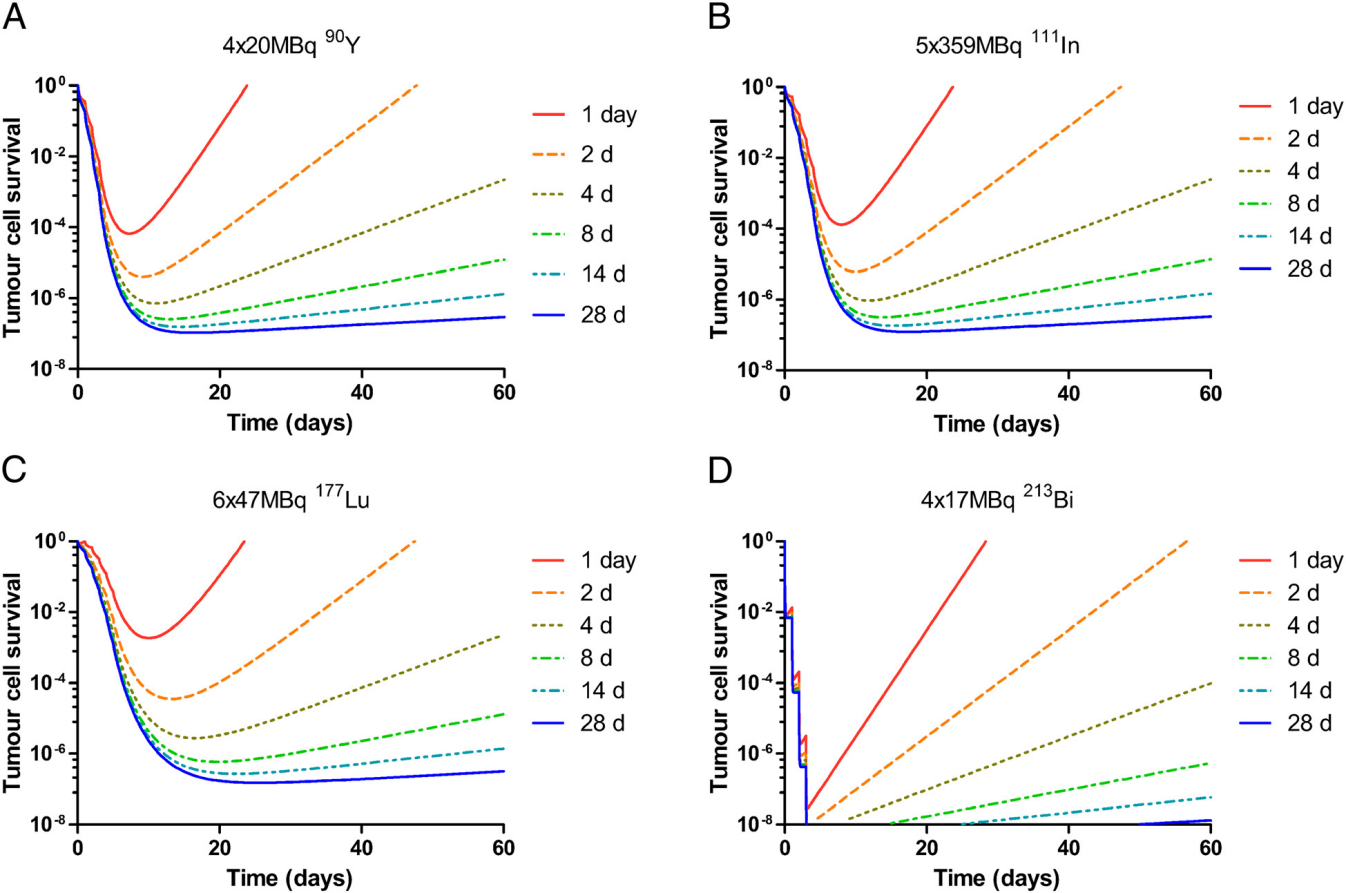
**Models for tumour growth curves.** After multiple administration of **(A)** ^90^Y, **(B)** ^111^In, **(C)** ^177^Lu or **(D)** ^213^Bi to a 300-mg tumour for different tumour doubling times. The same amount of peptide was used as indicated in Figure 4.

The reduction in tumour size as a function of time is shown in Figure 5 during a time period of 60 days. None of the tumours with a doubling time of more than 4 days grow back to their original size within this time period. The reduction in size is proportional to the radionuclide half-life and intervals (here 24 h) between the injections.

## Discussion

Based on studies with 12 CCK2 receptor-binding analogues, the biodistribution of the CCK2 analogue PP-F11 shows a very favourable uptake in CCK2 receptor-expressing tumours and relatively low renal retention, which makes it potentially suitable for therapeutic application. This study aimed to investigate its possibilities and hurdles for successful radionuclide therapy with this peptide analogue in mice with s.c. CCK2 receptor-expressing tumours. The particle range of the selected radionuclides has an important impact on the cure capability of the radioloabelled peptide for various sizes of tumours [3]. Since the receptor-mediated tumour uptake of radiolabelled PP-F11 shows a saturable relation with peptide mass, the maximum achievable specific activity is an important parameter in this respect. Furthermore, it has to be taken into account that the production method of radionuclides determines the amount that can be labelled to peptides. Therefore, the higher specific activities reachable with ^90^Y and ^213^Bi make both radionuclides very suitable for therapy of 200- to 300-mg tumours, using lower peptide mass and thus higher tumour uptake. Yttrium (^90^Y) is capable of controlling larger-sized (>200 mg) tumours, whereas ^213^Bi is capable of curing over the whole range of masses considered, but most optimal in the smallest sizes.

This receptor saturation effect has been observed previously with somatostatin receptor-targeted peptides, both in preclinical biodistribution studies [9], as in clinical use [27,28], and has also been observed even more prominently with bombesin analogues [29]. The limited number of receptors influences the tumour uptake of the peptide at escalating peptide doses. Pharmacokinetic compartment modelling can be used to correct for the molar concentration and receptor binding [11]. In this study, we used the one-site dissociation curve to describe the saturable uptake in the tumour and stomach. Saturation of tumour uptake in humans after PRRT with ^177^Lu-DOTA-octreotate has not been observed, whereas the receptor-mediated uptake in the liver and spleen did show a decrease by the therapeutic amount of peptide [30].

The α-emitter ^213^Bi shows ideal properties to cure small lesions, with most of its absorbed dose coming from its short half-life daughter ^213^Po. The RBE of ^213^Bi-PP-F11 is not known, but when the effects are comparable to the RBE found for ^213^Bi-DOTΑ-octreotate of 3.4 [26], its effect is tremendous. The absorbed dose to the normal organs such as the kidneys and stomach wall has not been considered in this evaluation, as kidney protection by blocking reuptake of the peptide can reduce the renal dose [31,32]. For all radionuclides, radiation-induced toxicity in normal organs will form the limiting amount of radioactivity. Late kidney damage will occur after cumulative doses of >27 Gy with ^90^Y-DOTA-octreotide [33]. Renal uptake of the PP-F11 peptide is hardly influenced by the injected peptide mass, contrary to the tumour uptake. Consequently, using a higher amount of peptides to increase the amount of radioactivity will lead to higher absorbed doses to the kidneys while the tumour absorbed doses are decreased due to receptor saturation. The stomach wall will show radiation-induced ulceration after 45-Gy fractionated external beam exposure [34]. For that reason, it is most probably not realistic to administer the huge amounts of radioactivity as suggested for ^111^In and ^177^Lu in the case of single-exponential clearance (Figure 3A).

Fractionation of the therapy is necessary for the practically achievable specific activities of the radionuclides considered. Fractionation will lead to higher cumulative absorbed doses in tumours and has been shown to lead to lower probability of renal toxicity in clinical application of radionuclide therapy [35]. Fractionation of therapy with ^177^Lu-DOTA-octreotate in rats with CA20948 tumours has been shown to reduce renal toxicity while maintaining the same tumour response as for the single fraction therapy [36]. This reduction in renal toxicity was also influenced by the time interval between the fractions; renal toxicity was significantly reduced for intervals of a week or longer compared to a 1-day interval. This inter-fraction recovery of renal damage is not fully explained by the repair mechanism within the LQ model [37]. In clinical practice, ^177^Lu-DOTA-octreotate is given in four fractions of 7.4 GBq with a 6-week interval, also in order to allow the bone marrow to recover [38].

The tumour growth rate has a large influence on the cure options using the longer half-life radionuclides. Tumours with a doubling time of less than 8 days show >50% reduction of the TCP for treatment using ^90^Y or ^111^In, and with ^177^Lu, this reduction sets in at doubling times below 14 days. Due to its short half-life, ^213^Bi is capable of controlling also fast-growing tumours. This growth effect is only of concern in preclinical studies with fast-growing tumour xenografts and hardly in the clinical setting in which tumour doubling times most often are longer than 14 days. Selective control of slower growing tumour subtypes could however induce a more aggressive type in any tumour regrowth. The concept of TCP may be a too strict criterion for cure, as for slower growing tumours, the regrowth may not be visible within the typical observation period of 2 to 3 months. A lag time without tumour growth and subsequent accelerated repopulation as observed in prolonged radiotherapy sessions has not been modelled [39]. The effect of repopulation after radionuclide therapy needs to be investigated further as it will strongly influence the cure rates for fast-growing tumours, e.g. with a lag time of 7 to 8 days, all growth effects would diminish when the therapy is completed within 1 week. The size of the tumour may not be completely representative for the number of viable cells within this volume. Doomed cells can form a part of the tumour volume in response to the radiation therapy, waiting to be cleared or encapsulated [40].

The basis for the radiobiological considerations is formed by the linear-quadratic model, which assumes that the tumour cells behave as cells in culture in response to radiation. The main LQ model parameters *α* and *β* are determined from external beam experiments. Response to radionuclide-induced doses is also very dependent on the repair process of sub-lethal damage. In external beam exposure, this repair takes place immediately after the dose given in a short time. With radionuclide-induced exposure, the repair is induced during the dose delivery and therefore the half-time value with which sub-lethally damaged lesions are repaired is very critical. In this work, this repair half-time was taken as 0.5 h, which is considered to be valid in general for tumours. Changing the repair half-time to 1 h increases the TCP by 10% to 20% for the beta-emitting radionuclides. Future work on radiobiological modeling for radionuclide therapy should focus on the repair mechanism and the influence of kinetics. The clearance kinetics is a dominant factor not only in the absorbed dose to the tumour, but also in the TCP by the projected dose rate. It is therefore of great importance to determine the kinetic pattern with more detail than just the two time points as taken in this study. Fractionation of radionuclide therapy should be studied on the basis of the absorbed doses given, taking the radiation sensitivity, the repair of sub-lethal damage and recovery by repopulation into account.

## Conclusions

Receptor-mediated uptake of the CCK2 compound PP-F11 in tumours shows a steep saturation effect with increasing amounts of peptide.

The specific activities achievable for radiolabelling peptides limit the absorbed dose in tumours, as higher activities go parallel with higher peptide amounts, which might considerably reduce the tumour uptake.

Multiple injections are preferred to obtain an absorbed tumour dose in the order of 60 Gy.

For the larger xenografts (>200 mg), ^90^Y is the preferable radionuclide, as its high specific activity allows the use of low peptide amounts, although it lacks the capability of curing smaller-sized tumours.

For tumour xenografts in almost the whole size range (<500 mg), ^213^Bi shows high TCPs, taking an enhancement by increased RBE from its α-radiation into account.

The influence of the clearance kinetics is the largest with ^177^Lu, whereas ^213^Bi is most strongly influenced by the uptake kinetics.

Ongoing growth of the tumour during the absorbed dose build-up reduces the TCP for longer half-life radionuclides and has hardly any influence on the cure options for ^213^Bi.
